# Enhanced Antioxidant and Antibacterial Properties of Polybutylene Adipate-Terephthalate/Curcumin Composite Films Using Surface-Modified Cellulose Nanocrystals

**DOI:** 10.3390/polym17070830

**Published:** 2025-03-21

**Authors:** Hashimu Juma, Cunshi Zhao, Qingbo Wang, Yunfeng Guo, Xinyan Fan, Wuming Fan, Linlin Zhao, Jiayi Sun, Dong Wang, Yonggui Wang

**Affiliations:** Key Laboratory of Bio-Based Material Science and Technology, Ministry of Education, College of Material Science and Engineering, Northeast Forestry University, Hexing 26 Road, Harbin 150040, China; nalupihashimu96@gmail.com (H.J.); 134035894817@163.com (C.Z.); qingbo.wang@nefu.edu.cn (Q.W.); 18814665913@163.com (Y.G.); c377648@nefu.edu.cn (X.F.); fl10316@163.com (W.F.); zll20000211@163.com (L.Z.); sunjiayi021230@163.com (J.S.); dong.wang@nefu.edu.cn (D.W.)

**Keywords:** cellulose nanocrystals, curcumin, poly (butylene adipate-co-terephthalate), composite films

## Abstract

Polybutylene adipate-terephthalate (PBAT) offers a convincing ecological alternative to the traditional fossil-based plastics due to its biodegradability and robust mechanical properties. The objective of this study is to develop PBAT-based bio-composite films through incorporating functionalized cellulose nanocrystals (CNC) and curcumin (CUR). In order to improve the interfacial compatibility with the PBAT matrix and co-doping with CUR, CNC was modified using dodecyl succinic anhydride (D_x_CNC). In this ternary bio-composite system, CUR functioned as a bio-based antioxidant and antimicrobial agent. The presence of CUR also provides excellent UV-shielding properties, whereas the D_x_CNC effectively enhances the controlled release of CUR. The synergistic effect between D_x_CNC and CUR in boosting antimicrobial properties, with the inhibition values for *E. coli* and *S. aureus* reached 1.82 log CFU/cm^2^ and 2.12 log CFU/cm^2^, respectively. These findings indicate D_x_CNC/CUR/PBAT ternary composite films as a promising material for eco-friendly packaging products.

## 1. Introduction

Interest in environmentally friendly polymers has grown due to the environmental challenges caused by non-biodegradable plastics, including waste accumulation, pollution, and the depletion of oil supplies [1]. As awareness of environmental sustainability increases, numerous countries are imposing restrictions on petroleum-derived and non-biodegradable materials, especially disposable plastics, aiming to promote sustainable practices and address the ecological consequences of plastic waste [2]. Biodegradable polymers are considered a promising alternative to replace non-biodegradable plastics, attributed to their eco-friendly and less toxic characteristics [3]. Nowadays, the development of biodegradable plastic materials made from polymers like poly (butylene adipate-co-terephthalate) (PBAT), polylactide (PLA), and polyethylene terephthalate (PET) has attracted increasing attention. PBAT, an aliphatic-aromatic polyester that combines the characteristics of both polybutylene adipate and polybutylene terephthalate, stands out as a promising biodegradable polymer due to its excellent properties, including superior thermal stability, biocompatibility, flexibility, ease of processing, and transparency [4,5,6]. Furthermore, PBAT has gained considerable research attention for its biodegradability and environmentally friendly qualities. However, the limited antioxidant and antibacterial properties of PBAT limit its performance in high-performance packaging applications.

Incorporating inorganic nanoparticles, such as ZnO, Ag, TiO_2,_ and SnO_2_, is a method to enhance the antibacterial effects of PBAT-based composites [7,8,9,10]. The inorganic nanoparticles could effectively disrupt bacterial cell membranes, generating reactive oxygen species (ROS), and release metal ions to fight against antimicrobial resistance [11]. However, applying inorganic nanoparticles in packaging applications might raise concerns about their toxicity to human health and the associated high costs of the process [12,13]. Research also investigates using natural biopolymers, such as chitosan, lignin nanoparticles, and oxidized starch, incorporated PBAT composite films for food contact packing applications [14,15,16]. However, the antibacterial performance is largely dependent on the surface chemistry of the natural polymers [17]. Thus, a composite should either doping at least 20% of natural polymers or carefully coating a thin layer of natural polymer on the film [14]. Recently, curcumin (CUR), a naturally occurring bioactive small molecule compound extracted from *Curcuma longa* (turmeric), has gained attention for its non-toxic nature and hydrophobic phenolic structure. Moreover, research has shown that CUR, with its intrinsic structural characteristics, possesses antioxidant, UV-blocking, anti-inflammatory, and antimicrobial properties, thus significantly expanding its potential application scenarios, including biopolymer-based films, packaging, and biomedical devices [18,19,20]. CUR also present versatility and can be incorporated into composites with various biopolymers, such as starch, chitin, gelatin, and PLA, boosting their antioxidant and antibacterial performance as food packaging materials [21,22,23,24]. In previously reported studies, CUR-incorporated PBAT composite film was proposed to have potent antioxidant activity [25]. However, with the incorporation of CUR content, the CUR/PBAT composite film showed a decrease in mechanical performance, including strength, flexibility, and elasticity. This might be due to the poor dispersion of CUR and the disruption of the PBAT matrix. Moreover, the as-prepared CUR/PBAT (1% CUR content) composite film presents very limited antibacterial properties. The poor antibacterial properties are likely due to the dense PBAT network that affects the release of CUR, hindering its interaction with bacteria at the surface of the composite film. Thus, 1% of CUR in PBAT is not sufficient to guarantee antibacterial performance. Therefore, a material is required that can improve the compatibility between CUR and PBAT, preventing a significant decline in mechanical performance while also serving as a carrier for CUR to facilitate its better release and enhance its antimicrobial effects.

Cellulose nanocrystals (CNC) that are derived from cellulose are considered highly promising nanofillers due to their ability to significantly enhance the performance of various biopolymer systems [26]. CNC are rod-like structures with diameters ranging from 2 to 20 nm and lengths from 100 to 500 nm. They feature a high aspect ratio and a large surface area (about 150 m^2^/g) [27]. Meanwhile, CNC possess a crystalline structure that provides a high modulus of around 120–150 GPa. Thus, CNC is considered an ideal reinforcing agent for the PBAT network and a carrier for CUR [28]. However, the surface of CNC is abundant in hydroxyl groups, making them highly hydrophilic. This results in poor compatibility with the highly hydrophobic PBAT. Recent studies on PBAT/CNC nanocomposites have primarily focused on their compatibility and the role of CNC in the PBAT matrix by examining variables like CNC content, surface modification, and composite fabrication methods [29]. Octadecyl isocyanate-modified CNC/PBAT composite was proposed and fabricated using an internal melt mixer. However, CNC agglomeration in the PBAT matrix was observed when CNC content was beyond 3 wt% [30]. Another study proposed phenyl butyl isocyanate-modified CNC/PBAT composite fabricate using a twin-screw extruder and observed modest enhancements in elastic modulus with up to 10 wt% CNC [31].

Herein, we propose a ternary PBAT-based bio-composite film consisting of functionalized CNC and CUR with enhanced antioxidant and antibacterial properties. To improve the compatibility within the PBAT matrix, we modified the CNC surface with dodecyl succinic anhydride (D_x_CNC). Through comprehensive characterization, we proved the surface modification of D_x_CNC could not only distribute evenly within the PBAT but also prevents CUR aggregation. In addition, we also evaluate the influence of D_x_CNC concentration on the physical properties of the bio-composite. Most importantly, we demonstrate that the synergistic effect between D_x_CNC and CUR could significantly boost antimicrobial properties, with the inhibition values of *E. coli* and *S. aureus* reaching 1.82 log CFU/cm^2^ and 2.12 log CFU/cm^2^, respectively. In the end, this study highlights how the synergistic combination of D_x_CNC and CUR enhances the functional characteristics of PBAT composite, making it a promising candidate for high-performance, biodegradable materials for food and biomedical packaging and other eco-friendly applications. This work contributes to advancing the development of biopolymer-based materials with improved performance and sustainability.

## 2. Materials and Methods

### 2.1. Materials

The PBAT used in this work was a commercial grade of PBAT (Ecoflex^®^ C1200) in pellet form, supplied by BASF (Ludwigshafen, Germany). The wood powder was purchased from Harbin Yongxu Plastic Industry Co., Ltd., Harbin, China. 4-Dimethylaminopyridine (DMAP), 3-(1-dodecen-1-yl) dihydro-2, 5-furandione (DDSA), anhydrous N, N-dimethylformamide (DMF), and CUR were purchased from Shanghai Maclean Biochemical Technology Co., Ltd., Shanghai, China. 1,1-Diphenyl-2-picrylhydrazyl radical (DPPH) was purchased from Shanghai Aladdin Chemistry Co., Ltd., Shanghai, China. Ammonium hydroxide solution was purchased from Tianjin Tianli Chemical Reagent Co., Ltd., Tianjin, China. Anhydrous ethanol, tetrahydrofuran (THF), and dichloromethane (DCM) were purchased from Tianjin Fuyu Chemical Reagent Co., Ltd., Tianjin, China.

### 2.2. Preparation and Surface Modification of CNC

CNC was synthesized through HCl hydrolysis of purified cellulose, a widely established method for obtaining nanocellulose, as outlined in the Supporting Information. To modify the CNC surface, 1 g of dried CNC was dispersed in 20 mL of anhydrous DMF via ultrasound for 1 h. The mixture was then heated to 80 °C, followed by the addition of DDSA and DMAP in fixed proportions. After a 4 h reaction, the system was cooled, precipitated in ethanol, and centrifuged. The precipitate was purified through repeated washing with a THF-ethanol mixture and centrifugation to remove residual reagents. The samples were dried to obtain D_x_CNC, with the molar ratios of total hydroxyl content of CNC to DDSA being 1:1, 1:2, and 1:3, respectively, designated as D_1_CNC, D_2_CNC, and D_3_CNC. The amount of DMAP added was 5 wt% of DDSA.

### 2.3. Preparation of D_x_CNC/PBAT Composite Films

Solvent casting was used to obtain the D_x_CNC/PBAT composites. A 10 wt% D_x_CNC suspension was prepared in DCM, while 1 g of PBAT was dissolved separately in 15 mL of DCM. D_x_CNC was added to the PBAT solution in contents of 0%, 1%, 3%, 5%, and 7% by PBAT mass. The mixture was stirred for 5 h at ambient temperature, poured into Petri dishes, and allowed to evaporate for 12 h. Final drying occurred in a vacuum oven at 40 °C to remove residual DCM. Films were labeled based on D_x_CNC content: pure PBAT, 1% D_x_CNC/PBAT, 3% D_x_CNC/PBAT, 5% D_x_CNC/PBAT, and 7% D_x_CNC/PBAT.

### 2.4. Preparation of D_x_CNC/ CUR/PBAT Composite Films

Firstly, dissolve 10 mg of CUR in 15 mL of DCM, followed by the addition of 1%, 3%, 5%, and 7% D_x_CNC suspension (10 mg/mL) dispersed in DCM based on the mass of PBAT, respectively, and the mixture was stirred at 500 rpm for 24 h at ambient temperature to ensure uniform blending. Subsequently, 1 g of PBAT was added, and the mixture was stirred for an additional 5 h. The resulting mixture was cast onto a flat dish, evaporated at room temperature for 12 h, and fully dried at 40 °C. The films, labeled D_x_CNC/CUR/PBAT, were designated according to the D_x_CNC content (1%, 3%, 5%, and 7%).

### 2.5. Characterization

The morphology of CNC and D_x_CNC was characterized using transmission electron microscopy (TEM, JEM-2100, Tokyo, Japan). The chemical structure was analyzed by Fourier transform infrared spectroscopy (FTIR, Nicolette 6700, Waltham, MA, USA) and solid-state ^13^C nuclear magnetic resonance (NMR, AVANCE III, Berlin, Germany). The crystal structure of the samples was examined using X-ray diffraction (XRD, XRD-6100, Kyoto, Japan). The optical properties of PBAT and D_x_CNC/CUR/PBAT composite films were evaluated using a UV-Vis spectrophotometer (TU-1950, Beijing, China). The mechanical properties were tested at room temperature using a universal testing machine (C41.103, Xin SanSi Enterprise Development Co., Ltd., Shanghai, China). Rectangular specimens (40 mm × 10 mm) with an initial gauge length of 20 mm were subjected to tensile testing at a speed of 15 mm/min. The reported mechanical properties represent the average of five samples. The fractured surface morphology of the composite films was observed using a scanning electron microscope (SEM, EM-30 Plus, Daejeon, Republic of Korea), with samples being gold-coated prior to analysis to enhance conductivity.

The thermal properties of the composite films were evaluated using differential scanning calorimetry. The samples were first cooled from room temperature to −50 °C at a rate of 20 °C/min in a nitrogen atmosphere and held for 5 min. They were then heated to 200 °C at the same rate and maintained for 5 min, followed by re-cooling to −50 °C. The heating and cooling cycle was repeated. Data from the second heating cycle were used to analyze the thermal properties of the composite films.

The crystallinity (*X_c_*) of the composite films was calculated using following equation:

(1) $$X_{c}=\frac{{∆H}_{m}-{∆H}_{cc}}{∆H_{m}^{0}}\times \frac{100}{w}$$

where *X_c_* is the crystallinity of the sample; ∆*H_m_* is the enthalpy of fusion; ∆*H_cc_* is the enthalpy of cold crystallization; $∆H_{m}^{0}$ is the heat of PBAT when it is fully crystallized enthalpy (114 J/g); and *w* is the mass fraction of PBAT in the composite film.

The DPPH radical scavenging activity of the films was assessed using a previously reported method with minor modifications [32]. Film samples (100 mg) were immersed in 10 mL of a 0.004% (*w*/*v*) DPPH ethanol solution and kept under dark conditions for 24 h. A DPPH solution without film served as the blank control. After the incubation period, the absorbance of the solution at 517 nm was measured to determine the antioxidant activity of the film using the following equation:

DPPH radical scavenging activity (%) = (A_c_ − A_b_)/A_c_ × 100 (2)

where A_c_ is the absorbance of the control at 517 nm, and A_b_ is the absorbance of the films at 517 nm.

The antibacterial activity of pure PBAT, CUR/PBAT, and D_x_CNC/CUR/PBAT films was evaluated against *E. coli* and *S. aureus* using the modified ISO-22196 method [33]. A bacterial suspension (10^5^ to 10^6^ CFU/mL) was applied to 50 mm × 50 mm test films, and a 40 mm × 40 mm covering film was placed on top. The samples were incubated at 35 °C and ≥90% humidity for 24 h. After incubation, bacterial cells were recovered using phosphate-buffered saline, diluted, and plated on agar for colony counting. The log reduction of bacterial growth was calculated, allowing for the quantification of the films’ antibacterial effectiveness. At least three replicates were conducted to ensure reliability as follows [34]:

log reduction = log(A) − log(B)(3)

where A = the logarithmic mean of the number of viable bacteria recovered from control sample group after 24 h of inoculation, and B = the logarithmic mean of the number of viable bacteria recovered from test sample after 24 h of inoculation.

## 3. Results

### 3.1. Morphologies and Structures of D_x_CNC

**Figure 1:**
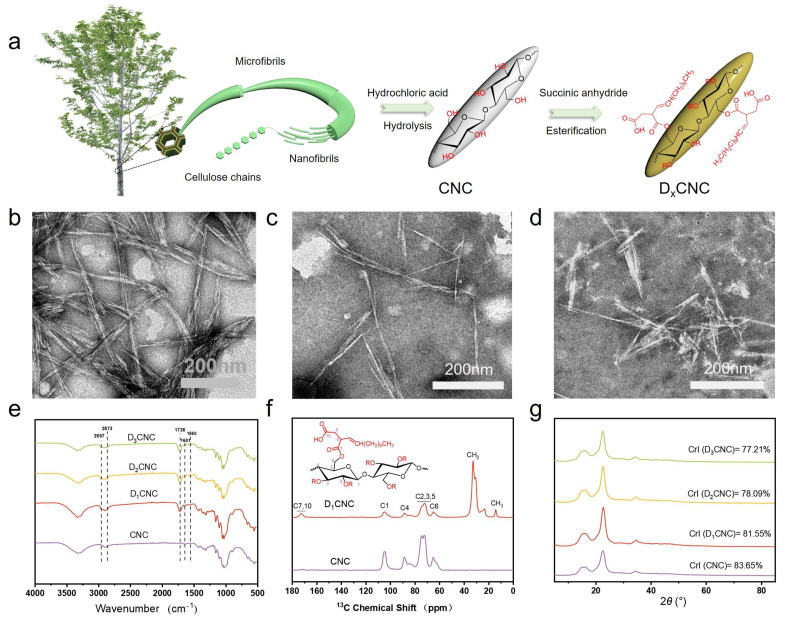
(**a**) Schematic diagram of D_x_CNC preparation process; (**b**–**d**) TEM images of (**b**) CNC, (**c**) D_2_CNC, and (**d**) D_3_CNC. (**e**) FTIR spectra of CNC and D_x_CNC; (**f**) Solid-state ^13^C NMR spectra of CNC and D_x_CNC; (**g**) XRD patterns of CNC and D_x_CNC.

The D_x_CNC was prepared as shown in Figure 1a. The CNC was extracted through acid hydrolysis, which effectively removed the amorphous regions from purified cellulose. TEM images confirmed their characteristic the rod-like nanocrystals with an average length of 100 to 200 nm, and diameters in the range of 5 to 20 nm (Figure 1b and Figure S1). This is consistent with the findings from other studies of CNC produced from acid hydrolysis [35]. After DDSA modification, as shown in Figure 1c,d, TEM images revealed notable morphological changes, including the aggregation of the D_x_CNC, suggesting successful esterification. Compared to CNC, the morphological contour of D_x_CNC appeared less defined, which is attributed to the alteration induced by the alkane chain layer on the surface of D_x_CNC.

FTIR and ^13^C NMR spectroscopy validated the successful modification of CNC, as shown in Figure 1e–f, respectively. The FTIR spectra of CNC and D_x_CNC have the same characteristic peaks, with some changes due to the esterification reaction. Compared to CNC, the modified D_x_CNC shows new absorption bands at 2957 cm^−1^ and 2873 cm^−1^, which corresponds to C-H stretching vibrations from the presence of DDSA, indicating the successful incorporation of alkyl chains from the anhydride. A distinct absorption peak at 1726 cm^−1^ was observed, which corresponding to the C=O stretching vibration of the ester bonds, confirming the esterification of the CNC [36]. New peaks at 1651 cm^−1^ and 1563 cm^−1^ were identified, which are attributed to the stretching vibrations of the C=C functional group and the carboxylate (COO^−^) group from DDSA. The results confirm that the esterification of CNC was effectively successful leading to the successful preparation of D_x_CNC. Furthermore, the solid-state ^13^C NMR spectra of CNC (Figure 1f) displayed a characteristic peak between 55–110 ppm, which corresponds to carbons in the dehydrated glucose of the unit cellulose I-type crystalline pattern. After esterification, new peaks appeared in the ^13^C NMR spectra of D_1_CNC indicating the introduction of ester groups into CNC, in particular a peak representing ester-bonded carbon at 173 ppm, while the signal peak of C4 in the crystalline phase at 88.5 ppm was markedly weakened in comparison to that of C4′ in the amorphous phase at 83.7 ppm (Figure 1f). These spectral changes from CNC to D_x_CNC, along with FTIR spectral changes, indicating successful chemical modification of CNC through esterification with DDSA.

Acid hydrolysis produces CNC with a significantly smaller diameter in comparison with other methods by selectively removing amorphous cellulose, resulting in higher crystalline cellulose [37]. The crystalline structure of CNC is critical to its reinforcing properties and should ideally be preserved following modification. Since esterification can potentially affect crystallinity, XRD measurements were performed to evaluate the effect of modification on crystallinity. The XRD patterns, as shown in Figure 1g, both CNC and D_x_CNC exhibit diffraction peaks at 2*θ* = 15.60°, 22.60°, and 34.52°, which are associated with the (101), (002), and (040) crystal planes of cellulose *I*-type structures [38]. These peaks illustrate how cellulose maintains an ordered structure due to hydrogen bonding between hydroxyl groups. The similarity in diffraction peaks for both CNC and D_x_CNC suggest that the crystalline structure is preserved after modification. CNC crystallinity was determined by using the Segal method, as reported in various studies [39]. The result of crystallinity value of CNC was found to be 83.65% at 2*θ* = 18.02° and 22.60°. In comparison, the crystallinity of D_1_CNC, D_2_CNC, and D_3_CNC decreased to 81.55%, 78.09%, and 77.21%, respectively. The decline in crystallinity for the esterified CNC is due to the reaction between DDSA and the hydroxyl groups on the CNC surface. This reaction weakens intermolecular hydrogen bonding, disrupting the ordered structure of the CNC and resulting in reduced crystallinity.

### 3.2. Optical Properties of D_x_CNC/CUR/PBAT Composite Films

**Figure 2:**
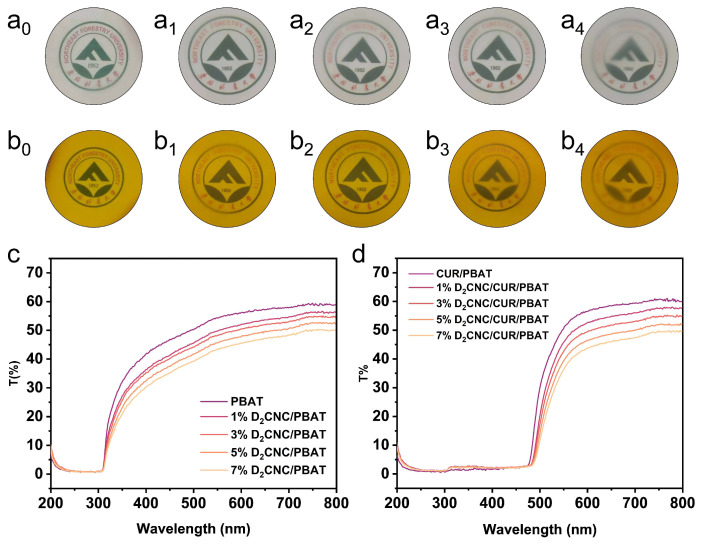
(**a**) Photos of (**a_0_**) PBAT film, (**a_1_**) 1% D_2_CNC/PBAT composite film, (**a_2_**) 3% D_2_CNC/PBAT composite film, (**a_3_**) 5% D_2_CNC/PBAT composite film, and (**a_4_**) 7% D_2_CNC/PBAT composite film; (**b**) Photos of (**b_0_**) CUR/PBAT composite film, (**b_1_**) 1% D_2_CNC/CUR/PBAT composite film, (**b_2_**) 3% D_2_CNC/CUR/PBAT composite film, (**b_3_**) 5% D_2_CNC/CUR/PBAT composite film, and (**b_4_**) 7% D_2_CNC/CUR/PBAT composite film; (**c**) Transmittance of PBAT and D_2_CNC/PBAT films; (**d**) Transmittance of CUR/PBAT and D_2_CNC/CUR/PBAT films.

The interaction between the surface of a polymer base and the nanofillers is the primary determinant of the effectiveness of composite materials. The D_x_CNC/PBAT and D_x_CNC/CUR/PBAT composite films were prepared using the solvent casting method with varying D_x_CNC content. Figure 2a,b and Figure S2 show the optical transparency of the PBAT nanocomposite films, indicating the quality of nanofiller dispersion within the polymer matrix and demonstrating that D_x_CNC and CUR were uniformly distributed throughout the processing. The neat PBAT and D_x_CNC/PBAT were translucent (Figure 2a), while films containing CUR exhibited a yellow color (Figure 2b), reflecting the natural color of CUR. The analysis of light transmittance reveals that the addition of D_x_CNC to the PBAT matrix results in a decrease in optical transparency, as shown in Figure 2c. A similar trend is observed in D_x_CNC/CUR/PBAT films (Figure 2d), indicating that higher D_x_CNC content adversely affects the dispersion quality and light transmission properties of these nanocomposite films. Pure PBAT had a transparency of 56.1% at 600 nm, attributed to its smooth and homogeneous structure, which is similar to the 56.5% observed for CUR/PBAT. However, the D_x_CNC/PBAT composite films with 7% CNC content had a transmittance of 43% at 600 nm (Figure 2c). This reduction in transparency can be associated with the different crystal structures and refractive indices of PBAT and D_x_CNC. Similar declines in transmittance with increasing CNC content have also been reported for other polymers, such as PLA and PVA, due to the refractive mismatch between the CNC and the polymer matrix [39]. In addition, both the CUR/PBAT and D_x_CNC/CUR/PBAT composite films exhibit a transmittance of 0% in the UV-absorbing region, providing particularly excellent UV-shielding properties. This phenomenon occurs because, when CUR is exposed to solar radiation, electronic excitations are induced within its molecular structure. In photochemical studies on UV radiation absorption, these electronic excitations can be efficiently converted into vibrational energy, particularly at the conjugated carbon-carbon double-bonded structure. This vibrational energy is subsequently dissipated to the surroundings as thermal energy, thereby preventing damage to the CUR structure and maintaining its molecular integrity [40,41]. On the other hand, under the influence of electronic excitation, the hydroxyl hydrogen atoms on the benzene ring of CUR are transferred to the carbonyl group. This hydrogen transfer process effectively absorbs the electronic excitation energy, playing a crucial role in stabilizing the CUR structure and mitigating potential damage from solar radiation [42]. The phenol structure and the α, β-unsaturated ketone structure of the conjugated double bonds in CUR are key factors enabling its strong UV light absorption. This excellent UV-blocking performance makes it highly suitable for applications in UV barriers for food packaging. When UV light penetrates packaging films, it can significantly degrade the quality and nutritional value of food by accelerating the decomposition of nutrients, ultimately leading to food spoilage. The D_x_CNC/CUR/PBAT composite films effectively address these issues by mitigating the harmful effects of UV radiation, thereby preserving the food’s quality and extending its shelf life.

### 3.3. Mechanical Properties of D_x_CNC/CUR/PBAT Composite Films

**Figure 3:**
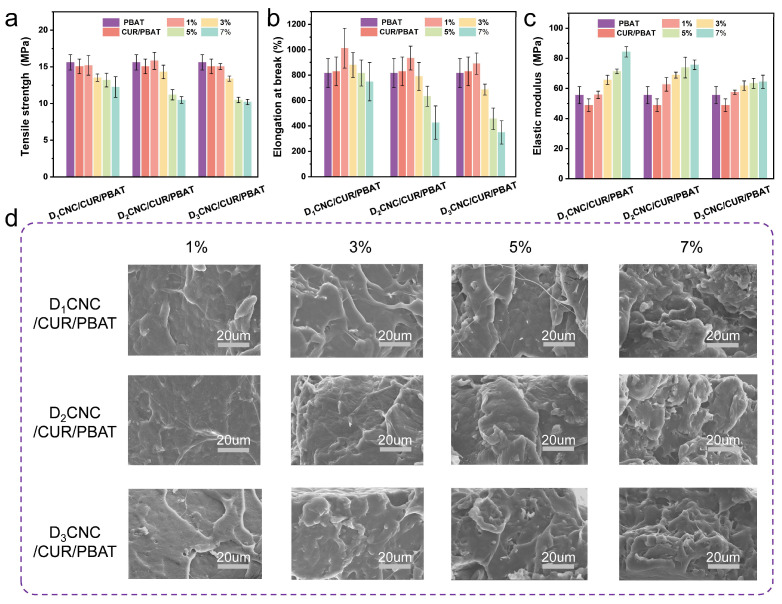
(**a**–**c**) Mechanical properties of pure PBAT and its nanocomposites: (**a**) tensile strength, (**b**) elongation at break, and (**c**) elastic modulus. (**d**) SEM images of the fractured cross-section of D_x_CNC/CUR/PBAT composite films.

Both D_x_CNC/PBAT and D_x_CNC/CUR/PBAT composites exhibited similar trends in mechanical performance, as shown in Figure 3 and Figure S3 and Table S1. The composite films exhibited a clear trend in mechanical performance. The neat PBAT film has a tensile strength, elongation at break, and modulus of elasticity of 15.61 MPa, 816.63%, and 55.69 MPa, respectively. With increasing D_x_CNC content, the tensile strength and elongation at break generally decreased, while the elastic modulus increased significantly. At low D_x_CNC content (1% D_2_CNC/CUR/PBAT), the composite achieved the highest tensile strength of 15.82 MPa and a notable elongation at break (935.18%). However, as the D_x_CNC content increased to 7% (7% D_2_CNC/CUR/PBAT), the tensile strength dropped to 10.48 MPa, and the elongation at break sharply declined to 426.27%, indicating reduced flexibility and increased brittleness. Although the dispersion of D_x_CNC in the PBAT matrix is relatively uniform, higher D_x_CNC content may restrict the mobility of PBAT molecular chains and disrupt the interactions between PBAT molecules, which ultimately contributes to reduced tensile strength and flexibility. Meanwhile, this trend suggests that excessive loading of D_x_CNC might lead to D_x_CNC aggregation within the PBAT matrix, resulting in stress concentration points and compromised mechanical strength [43]. Although the dispersion of D_x_CNC in the PBAT matrix is relatively uniform, higher D_x_CNC content may restrict the mobility of PBAT molecular chains and disrupt the interaction between PBAT molecules. The D_x_CNC/CUR/PBAT samples showed limited improvement in mechanical performance, likely due to the addition of CUR enhanced the filler–matrix interface that improved stress transfer between D_x_CNC and the PBAT matrix. We also observed an increase in the elastic modulus of the composite films, from 55.69 MPa (neat PBAT) to 75.70 MPa (7% D_2_CNC/CUR/PBAT), demonstrating the reinforcement effect of D_x_CNC. This improvement is attributed to CNC as a rigid nanofiller, which plays a significant role in reinforcing polymer matrices [44]. Meanwhile, the D_x_CNC is enriched with multifunctional surface groups, including hydroxyl and carboxyl groups, which lead to strong molecular interactions with the PBAT and CUR, and reinforce the composite film at low deformation. However, as deformation increases, the stress concentration points become the primary sites of mechanical failure, resulting in fracture and a sharp decline in the material’s tensile strength. We also observed that, at the same D_x_CNC content, composites with higher substitution degrees (D_3_CNC) generally exhibited lower mechanical properties compared to those with lower substitution degrees (D_1_CNC). This trend suggests that a higher degree of substitution might enhance D_x_CNC aggregation, leading to increased stress concentration points. Therefore, in the development of D_x_CNC/PBAT composites, the content and functionalization degree of D_x_CNC need to be optimized.

The fractured cross-section morphologies of PBAT-based composites, as displayed in Figure 3d for both D_x_CNC and CUR-reinforced samples, provide insight into the reinforcement mechanism at a microscopic level, as examined through SEM. Additionally, the distribution of fillers and interfacial adhesion are key factors affecting the mechanical properties of the composites. The microstructure of pure PBAT after stretching revealed a smooth and distinct fracture surface. At low D_x_CNC content, the composite films exhibit a smooth fracture surface with uniform fiber dispersion and no agglomeration, indicating effective interfacial bonding. This strong interaction facilitates stress dissipation and improves film performance [45]. This observation indicates that partial esterification of hydroxyl groups on the D_x_CNC surface reduces polarity, significantly enhancing D_x_CNC dispersion within the PBAT matrix and improving the performance of the composite film. However, at higher D_x_CNC concentrations, the composite cross-section shows increased voids and uneven cracks. At 1% and 3% D_x_CNC, small voids are observed, likely due to weak interfacial interactions between D_x_CNC and the PBAT matrix. This weak interaction leads to loosening of the PBAT molecular structure, facilitating crack formation near the interface, which may expand as D_x_CNC disperses throughout the matrix. Voids in the composites act as stress concentration points, reducing tensile strength. Increased D_x_CNC content leads to particle aggregation through hydrogen bonding, resulting in larger voids that further diminish tensile strength and adversely affect properties like elongation at break. Many fibers are pulled from the matrix under mechanical stress due to weak interfacial interactions, increasing fragility and lowering the overall mechanical properties of the bio-composites.

### 3.4. Thermal Properties of D_x_CNC/CUR/PBAT Composite Films

**Figure 4:**
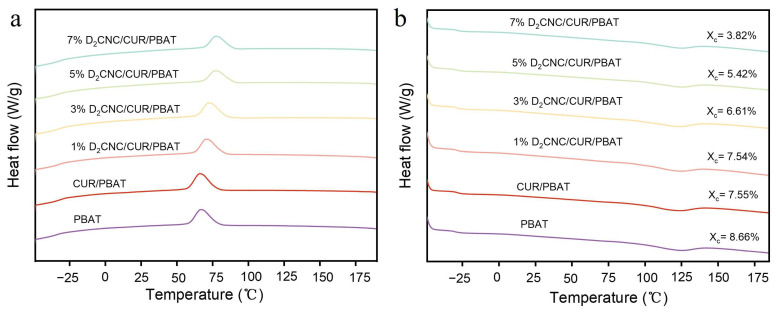
DSC curves of 2nd (**a**) heating and (**b**) cooling curves.

The thermal analysis highlights the effects of varying D_x_CNC content on key thermal parameters, including the glass transition temperature (T_g_), melting temperature (T_m_), cold crystallization temperature (T_cc_), and crystallinity of the nanocomposite films (Figure 4 and Table S2). From the data in Table S2, it can be concluded that the addition of CUR and D_x_CNC has no significant effect on T_g_. The T_cc_ of neat PBAT was 66.68 °C, representing the onset of crystallization during heating. While CUR minimally impacted T_cc_ (66.03 °C for CUR/PBAT), D_2_CNC caused a progressive increase in T_cc_, from 70.40 °C at 1% D_2_CNC to 77.11 °C at 7% D_2_CNC. This upward shift highlights the nucleating effect of D_2_CNC, which promotes crystallization at elevated temperatures by impeding chain mobility and delaying the rearrangement of polymer chains into ordered structures. The T_m_ of neat PBAT was observed at 123.66 °C, with a slight decrease to 122.54 °C upon the addition of CUR, possibly due to minor disruptions in the crystalline structure. Incorporating D_2_CNC demonstrated varying effects on T_m_; while 1% D_2_CNC caused a slight reduction to 123.22 °C, the 7% D_2_CNC sample showed a significant increase to 128.16 °C, indicating the enhanced stabilization of crystalline regions at higher D_2_CNC concentrations due to localized ordering effects.

The crystallinity of neat PBAT was 8.66%, reflecting its degree of molecular order. CUR reduced *X_c_* to 7.55%, likely due to its disruptive effect on PBAT’s crystalline domains. With increasing D_2_CNC content, the crystallinity decreased further, from 7.54% at 1% D_2_CNC to 3.82% at 7% D_2_CNC. This decline suggests that the rigid, dispersed nature of D_2_CNC nanoparticles interferes with the mobility and alignment of PBAT chains, thereby impeding the formation of large, well-ordered crystalline domains. Collectively, these results illustrate that while D_2_CNC enhances thermal stability and crystallization onset temperatures, it reduces the overall crystallinity of PBAT composites due to steric hindrance and restricted molecular mobility.

### 3.5. Antioxidant Properties of D_x_CNC/CUR/PBAT Composite Films

**Figure 5:**
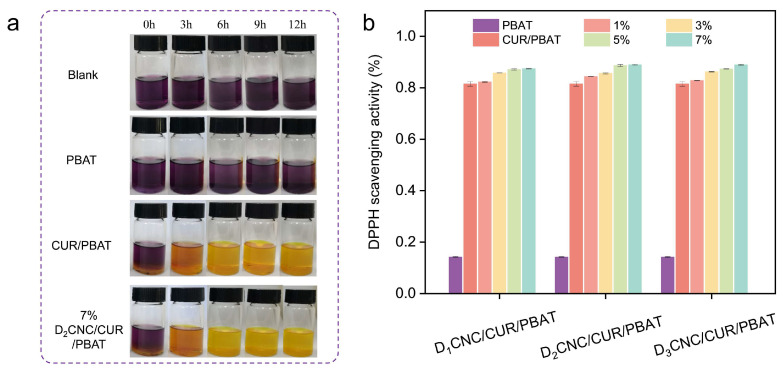
(**a**) Photos of the DPPH solutions after 0 h, 3 h, 6 h, 9 h, and 12 h of incubation with the composite films. (**b**) DDPH radical scavenging activities of PBAT, CUR/PBAT, and D_x_CNC/CUR/PBAT films.

The antioxidant properties of the composite films are assessed by measuring the DPPH free radical scavenging rate, as illustrated in Figure 5 and Figure S4. The analysis included neat PBAT, CUR/PBAT, and D_x_CNC/CUR/PBAT composite films to provide a comparative assessment of their free radical scavenging efficiency. The DPPH ethanol solution, initially dark purple, turns yellow when reduced by antioxidants capable of donating hydrogen atoms over varying time intervals, as shown in Figure 5a. Neat PBAT showed a minimal scavenging ability of 7.49%, while the CUR/PBAT composite demonstrated a rate of 75.34%, primarily due to the antioxidant properties of CUR, which neutralizes free radicals by donating hydrogen atoms from its phenolic hydroxyl groups [46,47].

The D_x_CNC/CUR/PBAT composites exhibited increased scavenging rates with higher D_x_CNC content. Specifically, the 1% D_x_CNC/CUR/PBAT film had a scavenging rate similar to that of CUR/PBAT. With the D_x_CNC content raised to 3%, 5%, and 7%, the scavenging rates increased to 82.13, 83.03, and 83.61%, respectively (Figure 5b). This tendency suggests that D_x_CNC effectively enables CUR release within the D_x_CNC/CUR/PBAT composite films. This enhancement is likely attributed to the ability of D_x_CNC to disrupt PBAT’s dense structure, thereby increasing the mobility and accessibility of CUR for interactions with free radicals. The hydrophobicity introduced by DDSA modification on D_x_CNC establishes a balance that promotes both the retention and controlled release of CUR, enhancing the composite’s antioxidant properties. Additionally, the remaining hydrophilic regions of D_x_CNC facilitate selective interactions with water molecules, further promoting CUR’s gradual release and sustained antioxidant activity [19]. The effectiveness of the CUR in the PBAT matrix is significantly impacted by both the structural characteristics of the PBAT and the contents of D_x_CNC.

### 3.6. Antibacterial Properties of D_x_CNC/CUR/PBAT Composite Films

**Figure 6:**
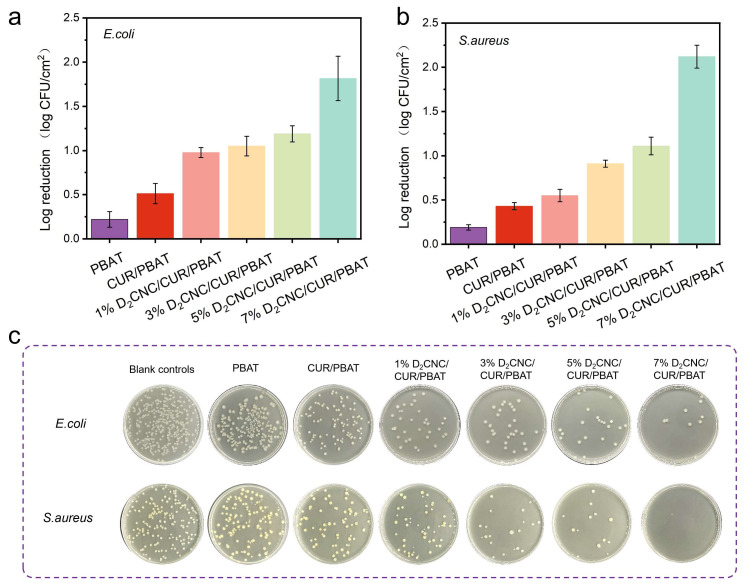
Antibacterial performance values of PBAT, CUR/PBAT, and D_2_CNC/CUR/PBAT composite films: (**a**) *E. coli*; (**b**) *S. aureus*; (**c**) Panel of photographs after 24 h incubation.

The antibacterial properties of D_x_CNC/CUR/PBAT composite films were evaluated against *S. aureus* (Gram-positive) and *E. coli* (Gram-negative), as illustrated in Figure 6. The results demonstrated that the incorporation of D_x_CNC and CUR into the PBAT matrix significantly enhanced the film’s antimicrobial performance. Neat PBAT films exhibited no measurable antibacterial activity, confirming their inherent inability to inhibit bacterial growth (Figure 6a,b). In contrast, CUR/PBAT films showed moderate antibacterial effects against both *E. coli* and *S. aureus*, reducing bacterial growth. This antibacterial activity is attributed to CUR’s established mechanism of disrupting bacterial cell division by binding to the essential FtsZ protein, thereby inhibiting its assembly and Z-ring formation, which are critical for bacterial cytokinesis [48,49].

The addition of D_x_CNC further enhances the antibacterial performance of the composite film. AFM imaging showed that D_x_CNC introduced a gradually rougher surface morphology (Figure S5), providing more surface support for CUR, enhancing the dispersibility of CUR in the PBAT matrix, disrupting the dense structure of the original PBAT molecular chains, increasing the contact area between CUR and bacteria, and improving its bioavailability. Furthermore, the evenly distributed D_x_CNC could modify the dense PBAT network and create more connected channels that enhanced the controlled release of CUR, thus presenting significant antibacterial performance compared to the previously reported PBAT/CUR films [25]. The improved release profile likely contributed to a more sustained and potent antibacterial effect, resulting in bacterial colony inhibition rates of approximately 1.82 log CFU/cm^2^ for *E. coli* and 2.12 log CFU/cm^2^ for *S. aureus*.

## 4. Conclusions

This study successfully developed and characterized PBAT-based bio-composite films incorporating D_x_CNC and CUR via solvent casting. The films were thoroughly evaluated to investigate the effects of D_x_CNC and CUR within the PBAT matrix. Structural and chemical analyses confirmed the effective modification of CNC with DDSA, ensuring its uniform distribution within the composite matrix. The bio-composite films demonstrated significant antioxidant and UV-shielding properties. With the addition of D_x_CNC, the bio-composite films demonstrated an enhanced elastic modulus with slightly decreased tensile strength. The films showed balanced performance with enhanced elasticity and flexibility, suggesting suitability for practical applications. Most notably, the films exhibited significant antibacterial activity, demonstrating the substantial inhibition of both *E. coli* and *S. aureus* and highlighting the synergistic effect of CUR and D_x_CNC in enhancing antimicrobial properties. These findings underscore the effectiveness of D_x_CNC and CUR as bio-based reinforcements for PBAT, not only enhancing its physical properties but also providing significant antibacterial performance. The results position these bio-composites, which offer antimicrobial properties, as an eco-friendly alternative to traditional petroleum-based polymers.

## Data Availability

The original contributions presented in this study are included in the article/Supplementary Material. Further inquiries can be directed to the corresponding author.

## Supplementary Materials

The following supporting information can be downloaded at https://www.mdpi.com/article/10.3390/polym17070830/s1: Synthesis scheme for purifying cellulose; morphological characterization of wood powder, purified cellulose, and CNC (Figure S1); Transmittance of D1CNC/PBAT, D3CNC/PBAT, D1CNC/CUR/PBAT, and D3CNC/CUR/PBAT composite membranes (Figure S2); Mechanical properties and cross-sectional SEM images of PBAT, CUR/PBAT, D1CNC/PBAT, D2CNC/PBAT, and D3CNC/PBAT (Figure S3); Composite films of DPPH radical scavenging capacity (Figure S4) and three-dimensional AFM surface morphology images (Figure S5); Mechanical performance of PBAT, CUR/PBAT, DxCNC/PBAT, and DxCNC/CUR/PBAT composite films (Table S1); DSC thermal parameter data of PBAT, CUR/PBAT, and D2CNC/CUR/PBAT composite films (Table S2). Reference [50] are cited in the Supplementary Materials.
