# Supplementary material for: Enhanced Antioxidant and Antibacterial Properties of Polybutylene Adipate-Terephthalate/Curcumin Composite Films Using Surface-Modified Cellulose Nanocrystals
Source: Polymers (Basel). 2025 Mar 21;17(7):830. doi: 10.3390/polym17070830 (PMC11990951; doi:10.3390/polym17070830)
Supplement: Supplementary file 1 [file polymers-17-00830-s001.zip › polymers-3518849-supplementary.pdf]

Supplementary Material

Enhanced Antioxidant and Antibacterial Properties of Polybutylene Adipate-Terephthalate/Curcumin Composite Films Using Surface-Modified Cellulose Nanocrystals

Hashimu Juma‡, Cunshi Zhao‡, Qingbo Wang, Yunfeng Guo, Xinyan Fan, Wuming Fan, Linlin Zhao, Jiayi Sun, Dong Wang, Yonggui Wang*

Key Laboratory of Bio-based Material Science and Technology (Ministry of Education), College of Material Science and Engineering, Northeast Forestry University, Hexing 26 Road, Harbin 150040, P, R. China

‡ Both authors contributed equally to this work

1. Preparation of CNC

CNC was prepared through HCl hydrolysis of purified cellulose, a method commonly used in other studies for nanocellulose (the preparation of purified cellulose adopts the traditional $NaClO_2$ chemical method [50]). HCl (120 mL, 12%) was added to purified cellulose (2 g) in a three-neck flask. After 2 h of sonication, the suspension was left to stand for 12 h to moisten the purified cellulose. Then, a catalyst, $FeCl_3$ (0.3 g), was added to the suspension, and the mixture was refluxed at 90°C for 5 h with magnetic stirring, after which the product was sonicated for 30 min, and the obtained suspension was washed five times via centrifugation (10000 rpm, 10 min) and transferred to a dialysis bag for 3 d. Finally, purified CNC was freeze-dried for further use.

2. Characterization

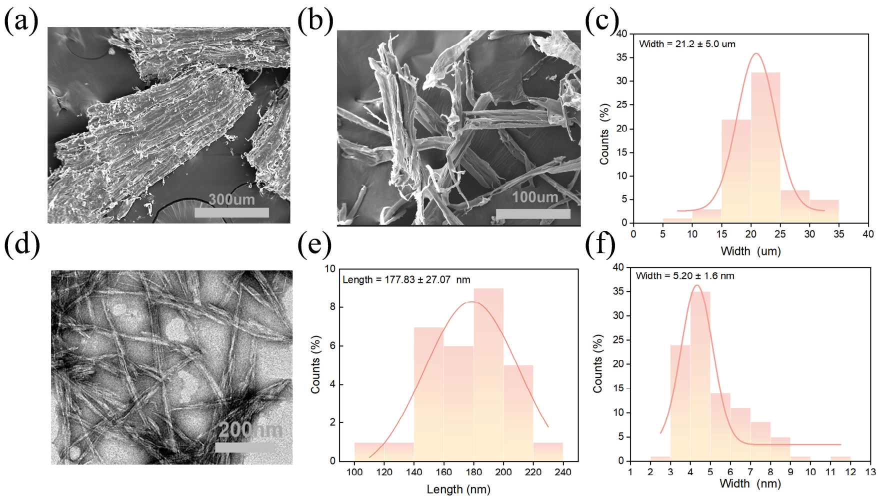

Figure. S1. (a) SEM image of wood powder; (b) SEM image of purified cellulose; (c) Width distribution histogram of purified cellulose; (d) TEM image of CNC; (e) Histogram of length distribution for CNC; (f) Width distribution histogram of CNC.

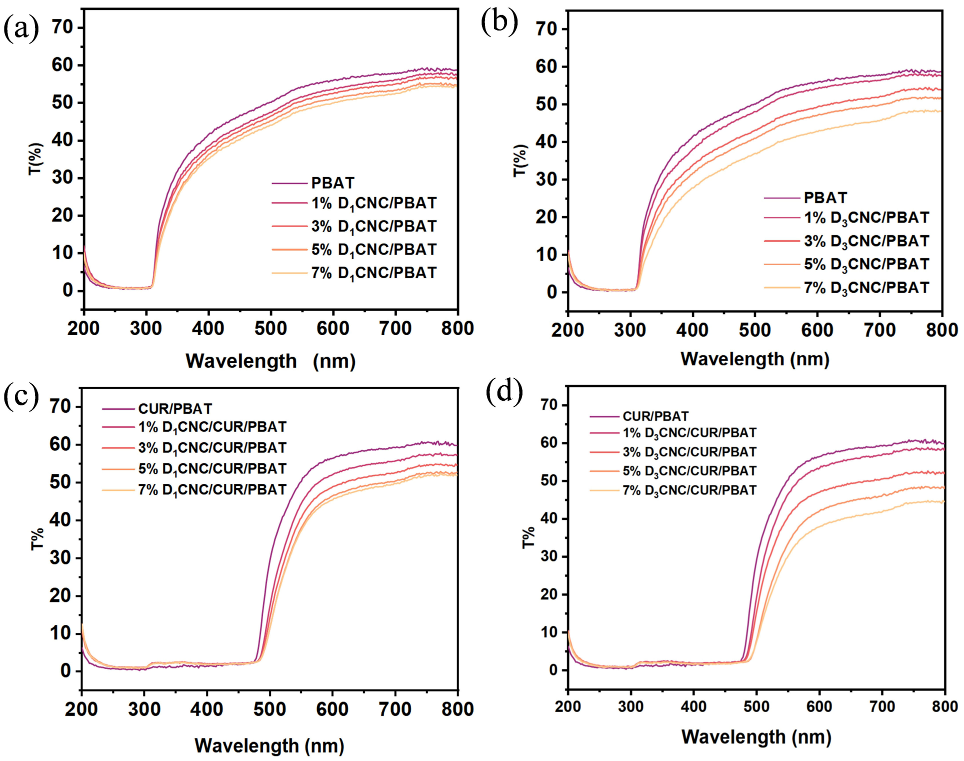

Figure. S2. Transmittance of (a) D_1CNC/PBAT composite films; (b) D_3CNC/PBAT composite films; (c) D_1CNC/CUR/PBAT composite films; and (d)

$D_3CNC/CUR/PBAT$ composite films.

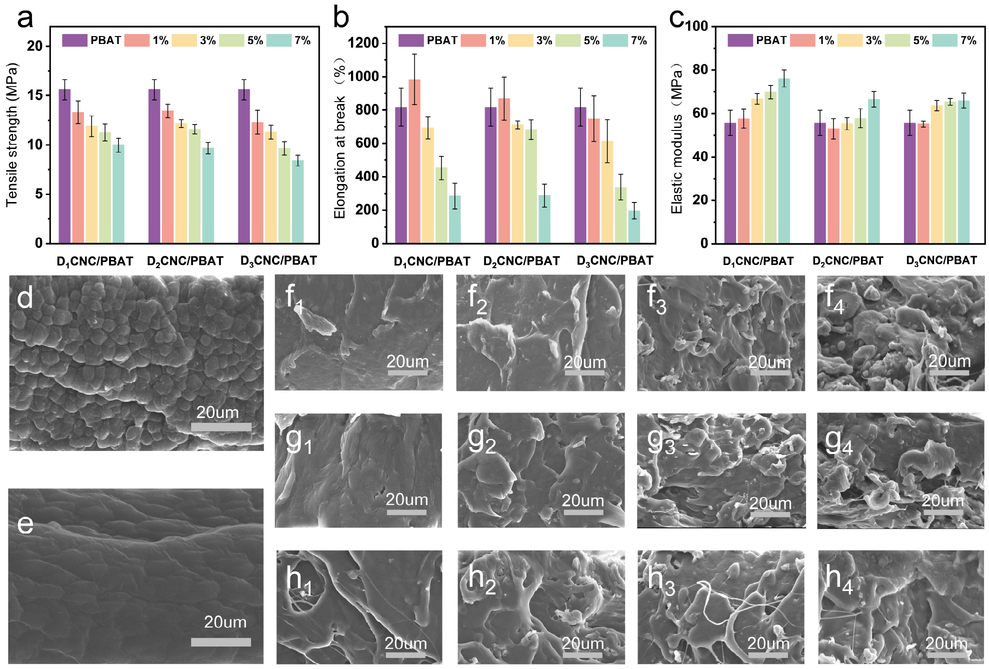

Figure. S3. Mechanical properties of pure PBAT and its nanocomposites: (a) Tensile strength; (b) Elongation at break; (c) Elastic modulus. SEM images of the fractured cross-section of (d) PBAT, (e) CUR/PBAT, and $D_xCNC/PBAT$ composite films with D_xCNC additions of 1%, 3%, 5%, and 7%, respectively: (f_1–f_4) $D_1CNC/PBAT$, (g_1–g_4) $D_2CNC/PBAT$, and (h_1–h_4) $D_3CNC/PBAT$.

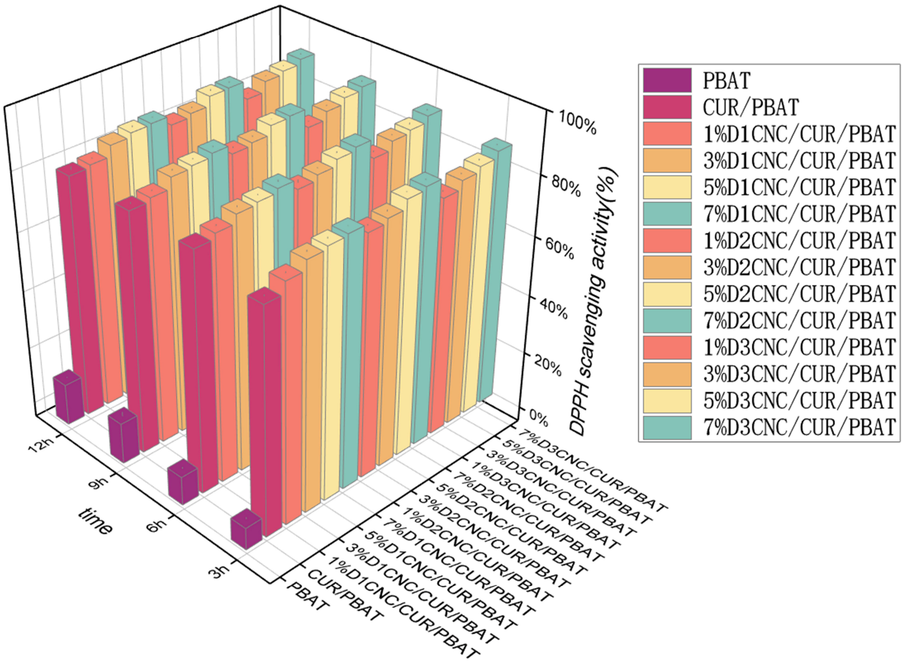

Figure. S4. DPPH radical scavenging capacity of the composite films.

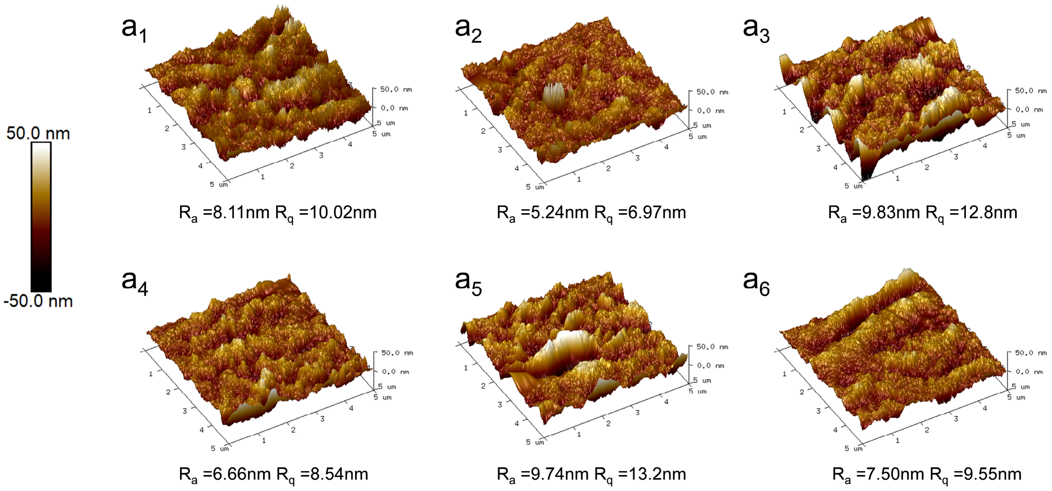

Figure. S5. (a_1-a_6) Three-dimensional AFM surface morphology images of the composite films: (a_1) PBAT; (a_2) CUR/PBAT; (a_3) 1% D_2CNC/CUR/PBAT; (a_4) 3% D_2CNC/CUR/PBAT; (a_5) 5% D_2CNC/CUR/PBAT; (a_6) 7% D_2CNC/CUR/PBAT.

Table S1. Mechanical performance of PBAT, CUR/PBAT, D_xCNC/PBAT, and

D_xCNC/CUR/PBAT composite films.

	Tensile strength (MPa)	Elongation at break (%)	Elastic modulus (MPa)
PBAT	15.61±1.04	816.64±113.50	55.58±5.75
CUR/PBAT	15.06±1.00	830.17±113.50	48.87±4.23
1% D_1CNC/PBAT	13.31±1.17	983.75±151.17	57.55±4.33
3% D_1CNC/PBAT	11.91±1.05	693.10±66.71	66.71±2.48
5% D_1CNC/PBAT	11.25±0.85	453.13±69.87	69.87±3.13
7% D_1CNC/PBAT	9.98±0.70	284.95±76.11	76.11±3.92
1% D_2CNC/PBAT	13.44±0.67	869.24±129.01	52.92±4.71
3% D_2CNC/PBAT	12.17±0.41	711.32±24.44	55.22±2.87
5% D_2CNC/PBAT	11.58±0.47	682.82±59.30	57.78±4.36
7% D_2CNC/PBAT	9.68±0.58	287.19±68.56	66.53±3.58
1% D_3CNC/PBAT	12.30±1.20	748.24±135.97	55.11±1.43
3% D_3CNC/PBAT	11.28±0.70	613.41±129.63	63.62±2.43
5% D_3CNC/PBAT	9.66±0.67	337.92±76.52	65.36±1.56
7% D_3CNC/PBAT	8.41±0.56	195.75±48.32	65.95±3.41
1% D_1CNC/CUR/PBAT	15.20±1.33	1012.36±156.60	55.82±2.40
3% D_1CNC/CUR/PBAT	13.52±0.50	880.42±97.97	65.75±3.03
5% D_1CNC/CUR/PBAT	13.20±0.94	816.86±102.17	71.36±1.59
7% D_1CNC/CUR/PBAT	12.25±1.40	748.75±151.46	84.34±3.47
1% D_2CNC/CUR/PBAT	15.82±1.16	935.18±93.78	62.75±4.50
3% D_2CNC/CUR/PBAT	12.17±0.92	790.26±110.54	68.90±1.95
5% D_2CNC/CUR/PBAT	11.20±0.71	633.97±79.17	73.90±6.89
7% D_2CNC/CUR/PBAT	10.48±0.45	426.27±130.92	75.70±3.21
1% D_3CNC/CUR/PBAT	15.05±0.40	890.87±83.37	57.49±1.45
3% D_3CNC/CUR/PBAT	13.39±0.36	686.34±43.43	61.82±3.18
5% D_3CNC/CUR/PBAT	10.49±0.37	456.72±84.64	63.45±3.32
7% D_3CNC/CUR/PBAT	10.21±0.35	349.70±91.48	64.48±4.36

Table S2. DSC thermal parameter data of PBAT, CUR/PBAT, and D_2CNC/CUR/PBAT composite films.

	T_g (℃)	T_{cc} (℃)	T_m (℃)	ΔH_{cc} (J/g)	ΔH_m (J/g)	X_c
PBAT	-29.30	66.68	123.66	13.436	9.8783	8.66%
CUR/PBAT	-28.38	66.03	122.54	14.137	8.6087	7.55%
1% D_2CNC/CUR/PBAT	-27.84	70.40	123.22	13.382	8.5954	7.54%
3% D_2CNC/CUR/PBAT	-27.99	72.34	122.56	12.167	7.5331	6.61%

5% D_2CNC/CUR/PBAT	-27.67	76.72	124.34	10.983	6.1759	5.42%
7% D_2CNC/CUR/PBAT	-27.34	77.11	128.16	10.862	4.3552	3.82%

References

[50] Chen, W.; Yu, H.; Liu, Y.; Hai, Y.; Zhang, M.; Chen, P. Isolation and characterization of cellulose nanofibers from four plant cellulose fibers using a chemical-ultrasonic process. *Cellulose* **2011**, 18 , 433-442.
